# Comparison of short‐term outcomes of robotic‐assisted and conventional laparoscopic surgery for rectal cancer: A propensity score‐matched analysis

**DOI:** 10.1111/ases.13075

**Published:** 2022-05-12

**Authors:** Takahiro Yamanashi, Hirohisa Miura, Toshimichi Tanaka, Akiko Watanabe, Takuya Goto, Keigo Yokoi, Ken Kojo, Masahiro Niihara, Kei Hosoda, Takashi Kaizu, Keishi Yamashita, Takeo Sato, Yusuke Kumamoto, Naoki Hiki, Takeshi Naitoh

**Affiliations:** ^1^ Department of Lower Gastrointestinal Surgery Kitasato University School of Medicine Sagamihara Japan; ^2^ Department of Upper Gastrointestinal Surgery Kitasato University School of Medicine Sagamihara Japan; ^3^ Department of General, Pediatric and Hepatobiliary‐Pancreatic Surgery Kitasato University School of Medicine Sagamihara Japan; ^4^ Division of Advanced Surgical Oncology, Department of Research and Development Center for New Medical Frontiers Kitasato University School of Medicine Sagamihara Japan; ^5^ Research and Development Center for Medical Education, Department Clinical Skills Education Kitasato University School of Medicine Sagamihara Japan

**Keywords:** propensity score‐matched analysis, rectal cancer, robotic‐assisted surgery

## Abstract

**Introduction:**

The advantages of robotic‐assisted laparoscopic surgery (RALS) for rectal cancer remain controversial. This study clarified and compared the short‐term outcomes of RALS for rectal cancer with those of conventional laparoscopic surgery (CLS).

**Methods:**

The records of 303 consecutive patients who underwent RALS or CLS for rectal adenocarcinoma between November 2016 and November 2021 were analyzed using propensity score‐matched analysis. After matching, 188 patients were enrolled in our study to compare short‐term outcomes, such as operative results, postoperative complications, and pathological findings, in each group.

**Results:**

After matching, baseline characteristics were comparable between groups. Although operative time in the RALS group was significantly longer than in the CLS group (*p* < 0.0001), the conversion rate to open laparotomy and the postoperative complication rate in the RALS group were significantly lower than in the CLS group (*p* = 0.0240 and *p* = 0.0109, respectively). Blood loss was comparable between groups. In the RALS group, postoperative hospital stay and days to soft diet were significantly shorter than those in the CLS group (*p* = 0.0464 and *p* < 0.0001, respectively). No postoperative mortality was observed in either group and significant differences were observed in resection margins and number of lymph nodes harvested.

**Conclusion:**

Robotic‐assisted laparoscopic surgery for rectal cancer was safe, technically feasible, and had acceptable short‐term outcomes. Further studies are required to validate long‐term oncological outcomes.

## 1 INTRODUCTION

Minimally invasive surgery (MIS) for rectal cancer has been widely adopted and performed under a variety of surgical settings. Findings of large randomized clinical trials (RCTs) have shown that conventional laparoscopic surgery (CLS) for rectal cancer has similar or better short‐term outcomes compared to open surgery (OS),^1^, ^2^ and similar long‐term oncological outcomes as OS.^3^, ^4^ However, the disadvantages of the CLS approach include poor visualization due to camera instability, limited dexterity of instruments with fixed tips, 2D view, and inadequate traction by the assistant. In terms of working in the deep narrow pelvis, performing total mesorectal excision (TME) using straight laparoscopic instruments that have a limited range of motion is also technically challenging. As a result, conversion rates in CLS for rectal cancer are as high as 9%–16%.^2^, ^5^, ^6^ Moreover, two large RCTs, which assessed circumferential resection margin (CRM) as pathological outcomes and as indicators of adequate surgical resection, showed higher positive CRM rates in CLS than in OS for rectal surgery, which may have been related to technical difficulties associated with the deep pelvis.^5^, ^6^

Robotic‐assisted laparoscopic surgery (RALS) is a relatively recent advance in MIS for rectal cancer. By employing articulated instruments, improved ergonomics, enhanced dexterity with tremor filtration, a stable 3D view, and motion scaling, RALS overcomes several of the limitations associated with CLS. Although the lower conversion rates of RALS relative to CLS were not established in the RCT,^7^ several studies have shown favorable outcomes in terms of the safety and feasibility of RALS for rectal cancer.^8^, ^9^, ^10^, ^11^, ^12^ However, the potential of RALS to overcome some of the limitations of CLS remains controversial, given the lack of evidence in the form of oncological and operative outcomes. Therefore, a retrospective study comparing short‐term outcomes of RALS and CLS for rectal cancer was conducted.

## 2 MATERIALS AND METHODS

### 2.1 Patients and data sources

**FIGURE 1:**
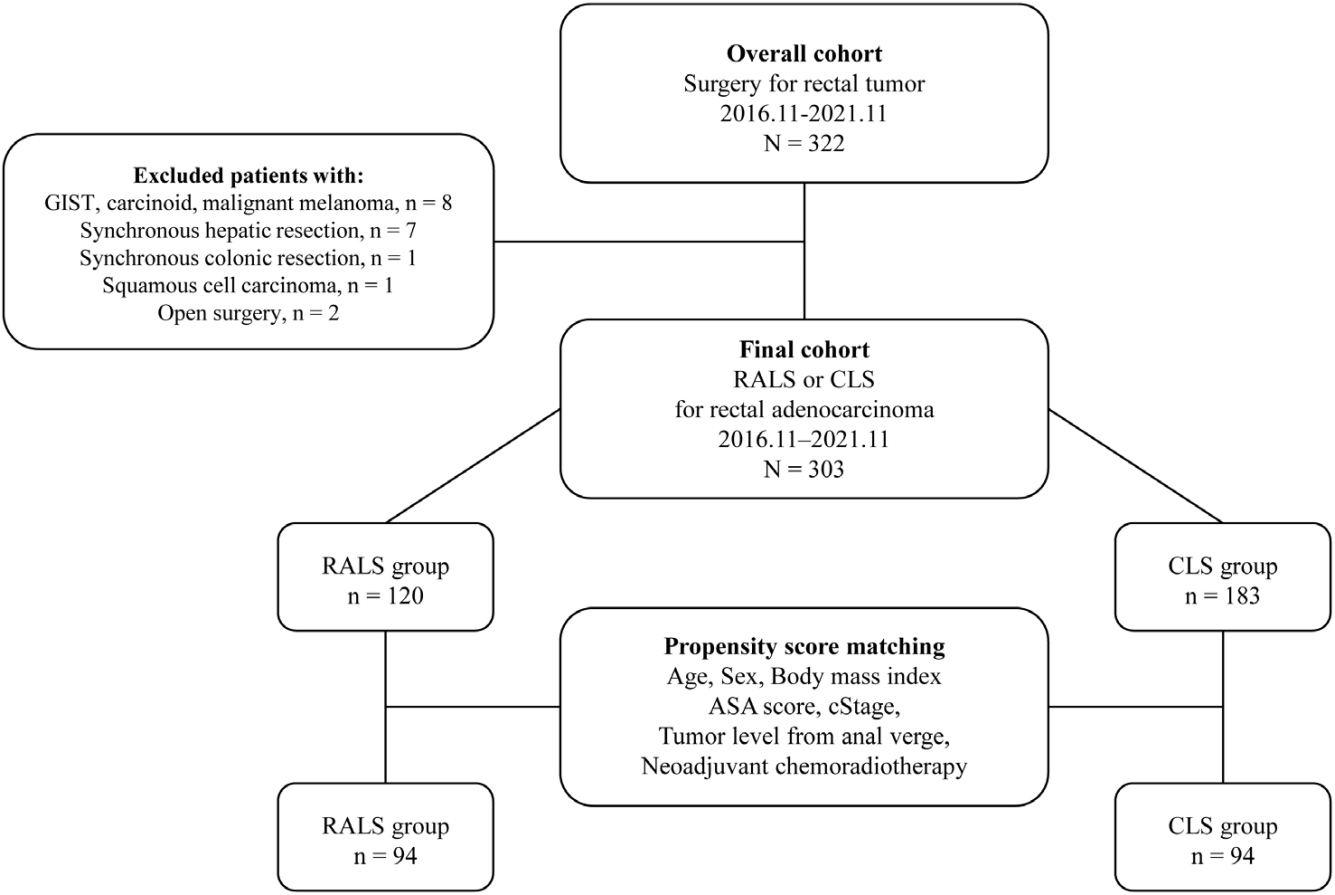
Study flow chart. RALS, robotic‐assisted laparoscopic surgery; CLS, conventional laparoscopic surgery; ASA, American Society of Anesthesiologists; GIST, gastrointestinal stromal tumor

This single‐center, nonrandomized, retrospective study compared RALS with CLS for rectal cancer. Patients who had primary rectal cancer with pathologically‐proven clinical stage I, II, III, or IV adenocarcinoma as defined by the Union for International Cancer Control (UICC) Tumor‐Node‐Metastasis Classification, 8th edition,^13^ were enrolled in this study. We retrospectively analyzed 303 consecutive patients who were treated for rectal cancer by RALS or CLS at Kitasato University Hospital, Japan, between November 2016 and November 2021. One hundred twenty patients were treated by RALS and 183 patients were treated by CLS (Figure 1). Patients were assigned depending on the surgeon's discretion and/or the availability of the Da Vinci Surgical System (Intuitive Surgical, Sunnyvale, CA, USA). Since we previously reported short‐term outcomes of 50 consecutive patients who underwent RALS for rectal cancer, some duplicate patients were included in this study.^14^

Patients' data used in this study were obtained from the medical records held at our hospital. For tumor location, the rectum was divided into the upper, mid, and lower rectum based on barium enema examination, colonoscopy, or pelvic magnetic resonance imaging (MRI). The upper‐ and mid‐rectum were defined as the lower border of the tumor located proximal to the peritoneal reflection. The upper‐ and mid‐rectum were defined as the center of the tumor located proximal and distal to the lower border of the 2nd sacral vertebra, respectively. The lower rectum was defined as the lower border of the tumor located distal to the peritoneal reflection. The degree of degeneration or necrosis of cancer cells was used to classify the treatment effect of neoadjuvant chemoradiotherapy (NCRT). A grade of 0 was assigned in cases when there was no response, while a grade of 3 was taken to indicate a complete response; the classification followed the Histopathological Response Criteria of the General rules for Clinical and Pathological Studies on Cancer of the Colon, Rectum and Anus edited by the Japanese Society for Cancer of the Colon and Rectum.^15^

The Institutional Review Board at our hospital approved this study (Approval No.: B21‐071). All patients were thoroughly informed about the surgical procedure and all of them provided written, informed consent.

### 2.2 Perioperative management

Some of the patients who were diagnosed with mid or lower rectal cancer with a clinical stage of cT3‐4 or N‐positive according to the UICC classification^13^ were treated following administration of NCRT. Undergoing NCRT was dependent upon the surgeon's discretion, patient's intention, or patient's performance status. NCRT was administered according to our institutional guidelines, as reported previously.^16^, ^17^ Before and after administering NCRT, a staging workup was performed using chest‐abdominopelvic computed tomography (CT), pelvic MRI, colonoscopy, and barium enema examination. Surgery was performed 8 to 10 weeks after NCRT completion.

Patients received the same standardized perioperative management protocol, including antibiotic prophylaxis, mechanical bowel preparation, thrombotic prophylaxis, analgesic care, and diet resumption. Oral intake was allowed after bowel movement returned, and was advanced to a soft diet gradually.

### 2.3 Surgical procedure

RALS was performed by three certified surgeons using the Da Vinci Si or Xi Surgical System configured as six‐ or five‐port systems, respectively. CLS was performed by seven surgeons certified by the Japan Society for Endoscopic Surgery and was configured using a five‐port approach. Patients were placed in a lithotomy position with the head tilted downward at 15–20° and the right side tilted downward at 15°. All procedures were performed in the colonic and pelvic phases. The colonic phase comprised inferior mesenteric artery and vein ligations and left‐sigmoid mesocolon mobilization, and the pelvic phase comprised pelvic dissection using TME or tumor‐specific mesorectal excision (TSME) principles.^18^ In patients in whom anterior resection (AR) was performed, the distal rectum was divided intracorporeally with a linear articulated endostapler loaded with a 45 mm or 60 mm cartridge. Bowel continuity was restored using the intracorporeal double‐stapling technique with a circular staple 25 mm in AR, or by transanal hand‐sewn suture in intersphincteric resection (ISR). All patients underwent curative standard resection with en bloc regional lymphadenectomy. Lateral lymph node dissection (LLND) was performed as required, when the short diameter of the lateral lymph node was swollen over 7 mm on preoperative CT. Lateral lymph nodes were removed around the common iliac vessel, internal iliac vessel, and obturator space, in the fat tissue outside the pelvic plexus. A diverting ileostomy was performed if necessary in AR and ISR.

### 2.4 Outcome parameters

In this study, the outcome parameters were blood loss, conversion rate to open laparotomy, days to soft diet, operative time, postoperative complications, postoperative hospital stay, postoperative mortality, reoperation, distal margin (DM), positive radial margin (RM), and number of lymph nodes harvested.

Conversion to open laparotomy from laparoscopic surgery was defined as the unintended extension of laparotomy beyond the incision necessary for specimen retrieval. Operative time was defined as the time between the initial skin incision and completion of wound closure. Postoperative complications, reoperation, and mortality were defined as events occurring during the postoperative hospital stay or within 30 days after surgery. The Clavien–Dindo (CD) classification was used to categorize postoperative complications.^19^ All such events were assessed by clinicians and documented in the database. To assess the quality of surgery, the pathological parameters of the surgical specimens were recorded, including DM, positive RM, and the number of lymph nodes harvested. Positive RM was defined as the actually exposed radial margin.

### 2.5 Statistical analysis

Descriptive data and continuous variables are presented as the mean and the standard deviation (SD) or the median and range, while categorical variables and the number of patients are presented as percentages. Prior to propensity score matching, the Mann–Whitney *U* test or Student's *t* test were used for continuous variables, and the Chi‐squared test was used for categorical variables. *P* < 0.05 was considered statistically significant. Propensity score matching was applied to adjust for differences in the baseline characteristics of patients and minimize the possibility of selection bias. First, multivariate logistic regression analysis was used to obtain the propensity score. To calculate the propensity score, the following seven covariates that could potentially affect the technical difficulty of performing rectal cancer surgery were included in the model: age, sex, American Society of Anesthesiologists score, body mass index, clinical stage, NCRT, and tumor level from the anal verge. The next step was 1:1 matching using a caliper coefficient of 0.2. Baseline characteristics, including operative results, postoperative complications, and pathological findings and covariates not entered into the model were then used to compare RALS with CLS. All statistical analyses were performed using JMP Pro statistical software (vr. 14, SAS Institute, Cary, NC, USA).

## 3 RESULTS

### 3.1 Baseline patient characteristics

Figure 1 shows a flow chart of this study. Of the 322 consecutive patients who underwent elective surgery for rectal tumor, two patients who underwent OS, nine patients with histologies other than adenocarcinoma (gastrointestinal stromal tumor, carcinoid, malignant melanoma, and squamous cell carcinoma), one patient who underwent synchronous colonic resection, and seven patients who underwent synchronous hepatic resection were excluded. Among the remaining 303 patients, RALS was performed on 120 patients (39.6%) and CLS was performed on 183 patients (60.4%). Table 1 shows the baseline characteristics of the overall cohort. Before matching, significant differences between RALS and CLS were observed in tumor level from the anal verge (*p* < 0.0001) and tumor location (*p* < 0.0001). After matching, 94 matched pairs were selected. Table 1 shows the demographic characteristics of the propensity score‐matched patients. With regard to baseline characteristics, the RALS and CLS groups were comparable.

**TABLE 1:** Baseline characteristics before and after matching

Characteristics	Overall (*N* = 303)			Propensity score‐matched pairs (*N* = 188)		
	RALS group (*n* = 120)	CLS group (*n* = 183)		RALS group (*n* = 94)	CLS group (*n* = 94)	
	median [range] or *n* (%) or mean ± SD		*p*	median [range] or *n* (%) or mean ± SD		*p*
Age, years	65 [21–86]	67 [16–89]	0.4136	65 [36–86]	66 [16–89]	0.9668
Sex			0.0864			0.6499
Male	80 (66.7)	104 (56.8)		61 (64.9)	58 (61.7)	
Female	40 (33.3)	79 (43.2)		33 (35.1)	36 (38.3)	
Body mass index, kg/m^2^	23.0 ± 3.2	22.8 ± 4.0	0.6702	23.2 ± 3.2	22.9 ± 3.6	0.5656
ASA score			0.1112			1.0000
1/2	107 (89.2)	151 (82.5)		81 (86.2)	81 (86.2)	
3	13 (10.8)	32 (17.5)		13 (13.8)	13 (13.8)	
Preoperative CEA level, ng/ml	3.4 [0.6–481]	3.1 [0.5–104]	0.2163	3.3 [0.8–481.0]	3.1 [0.6–60.5]	0.0864
Preoperative CA19‐9 level, U/ml	11.0 [3.0–856.0]	12.0 [1.0–373.0]	0.9968	11.0 [3.0–856.0]	10.5 [1.0–82.0]	0.5809
Tumor level from anal verge, mm	59 [5–200]	82 [10–200]	<0.0001	63 [10–200]	69 [10–161]	0.6680
Tumor location			<0.0001			0.2629
Upper rectum^a^	17 (14.2)	55 (30.1)		17 (18.1)	13 (13.8)	
Mid rectum^a^	28 (23.3)	59 (32.2)		23 (24.5)	33 (35.1)	
Lower rectum^b^	75 (62.5)	69 (37.7)		54 (57.5)	48 (51.1)	
cT category			0.5614			0.6363
T1	18 (15.0)	28 (15.3)		9 (9.6)	14 (14.9)	
T2	25 (20.8)	31 (16.9)		19 (20.2)	21 (22.3)	
T3	62 (51.7)	91 (49.7)		51 (54.3)	44 (46.8)	
T4	15 (12.5)	33 (18.0)		15 (16.0)	15 (16.0)	
T4a/T4b	12 (10.0)/3 (2.5)	19 (10.4)/14 (7.7)		12 (12.8)/3 (3.2)	6 (6.4)/9 (9.6)	
cN category			0.8332			0.4981
N0	73 (60.8)	113 (61.7)		56 (59.6)	56 (59.6)	
N1	32 (26.7)	44 (34.0)		26 (27.7)	21 (22.3)	
N2	15 (12.5)	26 (14.2)		12 (12.8)	17 (18.1)	
cM category			0.9792			0.7001
M0	116 (96.7)	177 (96.7)		90 (95.7)	91 (96.8)	
M1	4 (3.3)	6 (3.3)		4 (4.3)	3 (3.2)	
cStage^c^			0.8992			0.8664
I	39 (32.5)	54 (29.5)		26 (27.7)	30 (31.9)	
II	34 (28.3)	59 (32.2)		30 (31.9)	26 (27.7)	
III	43 (35.8)	64 (35.0)		34 (36.2)	35 (37.2)	
IV	4 (3.3)	6 (3.3)		4 (4.3)	3 (3.2)	
Neoadjuvant chemoradiotherapy	25 (20.8)	48 (26.2)	0.2827	25 (26.6)	23 (24.5)	0.7380

Abbreviations: ASA, American Society of Anesthesiologists; CA19‐9, carbohydrate antigen 19‐9; CEA, carcinoembryonic antigen; CLS, conventional laparoscopic surgery; RALS, robotic‐assisted laparoscopic surgery; TNM, Tumor‐Node‐Metastasis.

^a^ Upper‐ and mid‐rectum were defined as the lower border of the tumor located proximal to the peritoneal reflection.

^b^ Lower rectum was defined as the lower border of the tumor located distal to the peritoneal reflection.

^c^ Clinical stage, TNM classification of malignant tumors, eighth edition.

### 3.2 Operative results

Table 2 shows the operative results of the overall cohort (*N* = 303) and the propensity score‐matched cohort (*N* = 188). For the overall cohort, there was no conversion to open laparotomy and the CLS in RALS group, but conversion to open laparotomy was observed in five (2.7%) patients in the CLS group (*p* = 0.0240). Similarly, for the matched cohort the conversion rate to open laparotomy in the RALS group was significantly lower compared to that in the CLS group (*p* = 0.0402). The median operative time for the overall cohort was 346.5 min in the RALS group and 247 min in the CLS group. For both cohorts, the operative time of the RALS group was significantly longer than that of the CLS group (*p* < 0.0001). For both cohorts, the median number of days to soft diet was 1 day in the RALS group, which was significantly shorter than the 2 days in the CLS group (*p* < 0.0001). For the overall cohort, the median postoperative hospital stay was 10 days in the RALS group and 11 days in the CLS group. For the overall cohort and the matched cohort, the postoperative hospital stay was significantly shorter in the RALS group compared to the CLS group (*p* = 0.0464 and *p* = 0.0021, respectively). No significant difference in blood loss was observed between the groups.

**TABLE 2:** Operative results before and after matching

Characteristics	Overall (*N* = 303)			Propensity score‐matched pairs (*N* = 188)		
	RALS group (*n* = 120)	CLS group (*n* = 183)		RALS group (*n* = 94)	CLS group (*n* = 94)	
	*n* (%) or median [range]		*p*	*n* (%) or median [range]		*p*
Type of operation			0.0133			0.3625
High anterior resection	10 (8.3)	30 (16.4)		10 (10.6)	6 (6.4)	
Low anterior resection	77 (64.2)	115 (62.8)		61 (64.9)	61 (64.9)	
Intersphincteric resection	11 (9.2)	4 (2.2)		6 (6.4)	3 (3.2)	
Abdominoperineal resection	22 (18.3)	34 (18.6)		17 (18.1)	24 (25.5)	
Lateral lymph node dissection	11 (9.2)	14 (7.7)	0.6389	9 (9.6)	8 (8.5)	0.7993
Diverting ileostomy	76 (77.6^a^)	86 (57.7^a^)	0.0013	55 (71.4^a^)	46 (65.7^a^)	0.4555
Blood flow test using ICG	71 (72.5^a^)	19 (12.8^a^)	<0.0001	61 (79.2^a^)	10 (14.3^a^)	<0.0001
Operative time, min	346.5 [182–747]	247 [120–639]	<0.0001	342 [182–747]	254 [120–639]	<0.0001
Without lateral lymph node dissection	335 [182–634]	240 [120–530]	<0.0001	331 [182–634]	247 [120–517]	<0.0001
With lateral lymph node dissection	637 [354–747]	455.5 [256–639]	0.0020	637 [354–747]	477.5 [256–639]	0.0485
Blood loss, ml	5 [5–627]	5 [5–2267]	0.3137	5 [5–627]	7.5 [5–2267]	0.3189
Without lateral lymph node dissection	5 [5–627]	5 [5–2267]	0.2851	5 [5–627]	5 [5–2267]	0.3192
With lateral lymph node dissection	150 [5–539]	171.5 [5–900]	0.4763	100 [5–539]	159 [5–633]	0.4699
Transfusion	0 (0.0)	2 (1.1)	0.2506	0 (0.0)	2 (2.1)	0.1551
Conversion to laparotomy	0 (0.0)	5 (2.7)	0.0240	0 (0.0)	3 (3.2)	0.0402
Combined resection						
Other organs	1 (0.8)	11 (6.0)	0.0238	1 (1.1)	6 (6.4)	0.0541
Autonomic nerve system	1 (0.8)	3 (1.6)	0.5354	1 (1.1)	3 (3.2)	0.3013
Days to soft diet	1 [1–34]	2 [1–58]	<0.0001	1 [1–34]	2 [1–43]	<0.0001
Postoperative hospital stay, days	10 [6–62]	11 [6–94]	0.0464	10 [6–62]	12 [6–89]	0.0021

Abbreviations: CLS, conventional laparoscopic surgery; ICG, indocyanine green; RALS, robotic‐assisted laparoscopic surgery.

^a^ Data were analyzed in patients with anterior resection and intersphincteric resection.

For the overall cohort, despite significant differences in the operation type and the number of patients undergoing diverting ileostomy in the RALS and CLS groups, the findings were comparable for the matched cohort. For both cohorts, the frequency of intraoperative blood flow tests performed using indocyanine green (ICG) was significantly higher in the RALS group compared to the CLS group (*p* < 0.0001). In the overall cohort, combined resection of other organs was observed in one patient (0.8%) in the RALS group and 11 patients (6.0%) in the CLS group (*p* = 0.0238). There were no significant differences in terms of the rate for the combined resection of autonomic nerve system between groups in the overall and matched cohorts (*p* = 0.5354, *p* = 0.3013, respectively). Performing LLND was comparable between groups.

### 3.3 Postoperative complications

Table 3 shows a comparison of postoperative complications in both groups. For the overall cohort and the matched cohort, the incidence of moderate or severe complications (CD grade ≥ II) was significantly lower in the RALS group than in the CLS group (*p* = 0.0109 and *p* = 0.0018, respectively). For the overall cohort, anastomotic leakage above CD grade II was observed in 11 patients (11.2%) in the RALS group and 24 patients (16.1%) in the CLS group (*p* = 0.2817). On the other hand, for the matched cohort, anastomotic leakage in the RALS group tended towards being low compared to the CLS group (RALS 10.4% vs CLS 21.4%, *p* = 0.0658). For the overall cohort, urinary retention requiring placement of a urinary catheter (ie, above CD grade I) was observed in two patients (1.7%) in the RALS group and in 16 patients (8.7%) in the CLS group (*p* = 0.0108). Similarly, for the matched cohort, urinary retention in the RALS group was significantly lower than in the CLS group (RALS 1.1% vs CLS 10.6%, *p* = 0.0052). The incidence of other complications and reoperation was comparable in both cohorts of the two groups. No postoperative mortality was observed in either of the groups.

**TABLE 3:** Postoperative complications before and after matching

Characteristics	Overall (*N* = 303)			Propensity score‐matched pairs (*N* = 188)		
	RALS group (*n* = 120)	CLS group (*n* = 183)		RALS group (*n* = 94)	CLS group (*n* = 94)	
	*n* (%)		*p*	*n* (%)		*p*
Patient number, CD classification ≥ Grade II	20 (16.7)	54 (29.5)	0.0109	16 (17.0)	35 (37.2)	0.0018
Anastomotic leakage^a^	11 (11.2)	24 (16.1)	0.2817	8 (10.4)	15 (21.4)	0.0658
Urinary retention^b^	2 (1.7)	16 (8.7)	0.0108	1 (1.1)	10 (10.6)	0.0052
Small bowel obstruction	7 (5.8)	17 (9.3)	0.2759	6 (6.4)	10 (10.6)	0.2958
Wound infection	1 (0.8)	6 (3.3)	0.1658	1 (1.1)	4 (4.3)	0.1739
Ureter injury	1 (0.8)	1 (0.6)	0.7629	1 (1.1)	0 (0.0)	0.3160
Urethral injury	0 (0.0)	1 (0.6)	0.4173	0 (0.0)	0 (0.0)	n.a
Bleeding	0 (0.0)	2 (1.1)	0.2506	0 (0.0)	2 (2.1)	0.1551
Pelvic abscess	0 (0.0)	2 (1.1)	0.2506	0 (0.0)	2 (2.1)	0.1551
Wound dehiscence	0 (0.0)	2 (1.1)	0.2506	0 (0.0)	2 (2.1)	0.1551
Intestinal necrosis	1 (0.8)	1 (0.6)	0.7629	1 (1.1)	1 (1.1)	1.0000
Diarrhea	1 (0.8)	0 (0.0)	0.2161	0 (0.0)	0 (0.0)	n.a
Gastric ulcer	0 (0.0)	2 (1.1)	0.2506	0 (0.0)	2 (2.1)	0.1551
Brachial plexus neuropathy	0 (0.0)	1 (0.6)	0.4173	0 (0.0)	1 (1.1)	0.3160
CD classification ≥ Grade III	13 (10.8)	26 (14.2)	0.3910	11 (11.7)	16 (17.0)	0.2984
Reoperation	5 (4.2)	11 (6.0)	0.4827	5 (5.3)	9 (9.6)	0.2665
30‐day postoperative mortality	0 (0.0)	0 (0.0)	n.a	0 (0.0)	0 (0.0)	n.a

Abbreviations: CD, Clavien–Dindo; CLS, conventional laparoscopic surgery; n.a, not applicable; RALS, robotic‐assisted laparoscopic surgery.

^a^ Data were analyzed in patients with anterior resection and intersphincteric resection.

^b^ Data were analyzed in patients who had urinary retention with CD classification ≥ Grade I.

### 3.4 Pathological findings

Table 4 shows the pathological findings. In terms of the DM in both cohorts, no significant difference was observed between the RALS and CLS groups. Positive DM was not observed in any patient. However, for the matched cohort, positive RM tended to be observed in the RALS group compared to the CLS group (RALS 1.1% vs CLS 5.3%, *p* = 0.0970). For the overall and matched cohorts, the mean number of lymph nodes harvested in the RALS group and the CLS group was 14.8 and 16.3 (*p* = 0.1944), and 16.9 and 14.1 (*p* = 0.0377), respectively. Other pathological findings were comparable between both cohorts of the two groups.

**TABLE 4:** Pathological findings before and after matching

Characteristics	Overall (*N* = 303)			Propensity score‐matched pairs (*N* = 188)		
	RALS group (*n* = 120)	CLS group (*n* = 183)		RALS group (*n* = 94)	CLS group (*n* = 94)	
	Median [range] or *n* (%) or mean ± SD		*p*	Median [range] or *n* (%) or mean ± SD		*p*
Tumor size, mm	35 [5–90]	33 [3–140]	0.6051	35 [5–90]	32 [3–140]	0.3036
Histological grade			0.9743			0.7326
G1‐2 (pap/tub)^a^	114 (95.0)	174 (95.1)		89 (94.7)	90 (95.7)	
G3 (muc/por/sig)^b^	6 (5.0)	9 (4.9)		5 (5.3)	4 (4.3)	
Lymphatic invasion			0.5447			0.8762
Presence	38 (31.7)	52 (28.4)		31 (33.0)	30 (31.9)	
Absence	82 (68.3)	131 (71.6)		63 (67.0)	64 (68.1)	
Vascular invasion			0.9812			0.2324
Presence	70 (58.3)	107 (58.5)		53 (56.4)	61 (64.9)	
Absence	50 (41.7)	76 (41.5)		41 (43.6)	33 (35.1)	
Distal margin, mm	35 [10–90]	33 [3–114]	0.1349	35 [10–90]	30 [3–114]	0.4852
Positive radial margin	1 (0.8)	6 (3.3)	0.1658	1 (1.1)	5 (5.3)	0.0970
Number of lymph nodes harvested	16.3 ± 9.9	14.8 ± 9.0	0.1944	16.9 ± 9.9	14.1 ± 7.9	0.0377
Without lateral lymph node dissection	15.1 ± 8.9	14.6 ± 9.1	0.6463	15.5 ± 8.5	13.5 ± 7.9	0.1111
With lateral lymph node dissection	28.1 ± 12.2	18.1 ± 6.4	0.0149	29.6 ± 13.1	20.8 ± 5.7	0.0997
pT category			0.3700			0.7119
T0/Tis	4 (3.3)	11 (6.0)		4 (4.3)	7 (7.5)	
T1	32 (26.7)	37 (20.2)		22 (23.4)	22 (23.4)	
T2	28 (23.3)	39 (21.3)		19 (20.2)	22 (23.4)	
T3	48 (40.0)	75 (41.0)		42 (44.7)	34 (36.2)	
T4	8 (6.7)	21 (11.5)		7 (7.5)	9 (9.6)	
T4a/T4b	7 (5.8)/1 (0.8)	12 (6.6)/9 (4.9)		6 (6.4)/1 (1.1)	3 (3.2)/6 (6.4)	
pN category			0.5163			0.8929
N0	82 (68.3)	136 (74.3)		64 (68.1)	67 (71.3)	
N1	25 (20.8)	30 (16.4)		20 (21.3)	18 (19.2)	
N2	13 (10.8)	17 (9.3)		10 (10.6)	9 (9.6)	
c/pM category			0.7988			0.5157
M0	114 (95.0)	175 (95.6)		88 (93.6)	90 (95.7)	
M1	6 (5.0)	8 (4.4)		6 (6.4)	4 (4.3)	
pStage^c^			0.3490			0.8895
0/I	52 (43.3)	71 (38.8)		36 (38.3)	41 (43.6)	
II	26 (21.7)	59 (32.2)		24 (25.5)	23 (24.5)	
III	34 (28.3)	41 (22.4)		26 (27.7)	23 (24.5)	
IV	6 (5.0)	8 (4.4)		6 (6.4)	4 (4.3)	
pCR	2 (1.7)	4 (2.2)		2 (2.1)	3 (3.2)	
Treatment effect^d^			0.8269			0.9653
Grade 1	14 (56.0)	26 (54.2)		14 (56.0)	12 (52.2)	
Grade 2	8 (32.0)	18 (37.5)		8 (32.0)	8 (34.8)	
Grade 3	3 (12.0)	4 (8.3)		3 (12.0)	3 (13.0)	

Abbreviations: CLS, conventional laparoscopic surgery; n.a, not applicable; pCR, pathological complete response; RALS, robotic‐assisted laparoscopic surgery; TNM, Tumor‐Node‐Metastasis.

^a^ Papillary adenocarcinoma/well or moderately differentiated tubular adenocarcinoma.

^b^ Mucinous adenocarcinoma/poorly differentiated adenocarcinoma/signet‐ring cell carcinoma.

^c^ Pathological stage, TNM classification of malignant tumors, eighth edition.

^d^ Data were analyzed in patients who underwent neoadjuvant chemoradiotherapy.

## 4 DISCUSSION

In this study the oncological and operative outcomes between RALS and CLS were compared in patients with rectal cancer to evaluate the potential advantages of RALS. Propensity score‐matched analysis was performed in order to mitigate against selection bias and adjust for significant differences in baseline characteristics. Despite our extensive expertise in CLS for rectal cancer and our early experience with RALS for rectal cancer in this study the results reported here show that RALS had better or similar short‐term outcomes compared to CLS.

The conversion rate of MIS to open laparotomy reflects the technical complexity of the former. A low conversion rate to open laparotomy is clinically important because patients who were transferred from MIS to open laparotomy are more likely to develop postoperative complications and local recurrence.^20^, ^21^ Except for the excellent conversion rate of 1.2% reported for CLS at specialized centers,^1^ other large RCTs have shown that the conversion rate of CLS for rectal cancer typically ranged from 9% to 16%.^2^, ^5^, ^6^ Although the ROLARR trial failed to demonstrate the advantages of conversion to open laparotomy in RALS over CLS (8.1% vs 12.2%, *p* = 0.16),^7^ several meta‐analyses comparing RALS with CLS have demonstrated a lower conversion rate for RALS compared to CLS.^22^, ^23^, ^24^, ^25^ In this study, none of the patients in the RALS group were converted to open laparotomy; however, in the CLS group, five patients (2.7%) were converted to open laparotomy. Of the five patients who were converted from CLS to open laparotomy, four patients were converted due to tumor‐related factors, such as invasion into surrounding tissues that required combined resection. Our results have shown that the rate of conversion to open laparotomy in the RALS group was significantly lower than that in the CLS group for both cohorts. These results could be attributed to the difference in the number of combined resections, which were performed in patients in which invasion into surrounding tissues was observed. In the overall cohort, the rate of combined resection of other organs in the RALS group was significantly lower compared to that in the CLS group (*p* = 0.0238), and the rates of the patients with clinical T4b in the RALS group were less than those in the CLS group (2.5% vs 7.7%, not significant). It could be difficult to decide the excision line at the infiltrated surrounding tissues by using robotic arms, which have no sensation. There might be a possibility for the patients with clinical T4b who favorably underwent not RALS but CLS. Further prospective RCTs are necessary to evaluate whether the advantages of conversion to open laparotomy in RALS.

Several previous studies reported that, compared with CLS, RALS for rectal cancer was associated with significantly longer operative times.^11^, ^24^, ^25^, ^26^ The findings of our study were consistent with these previous studies, and the operative time for patients in the RALS group were significantly longer than that in the CLS group. In the present study the median operative time of patients who underwent RALS without LLND was 335 min and the median console time of TME or TSME procedures was 180 min (data not shown). The prolonged operative time required for RALS could be attributed to the time required to dock the robotic system, change instruments, and undock the system if the position of the patient had to be changed. Moreover, the present study included a learning curve period, which was previously reported to span from 20 to 75 cases.^27^, ^28^, ^29^, ^30^ After becoming proficient in operating the camera and manipulating the robotic forceps, as well as optimizing the settings of the robotic system, our operative time could likely be decreased. On the other hand, no significant differences in blood loss were observed between groups. Previous large RCTs comparing CLS with OS showed that median blood loss was 100–200 ml in the CLS group.^1^, ^2^, ^6^ In the present study, median blood loss in the overall cohort of the CLS group was 5 ml, which is extremely low compared to previous reports. Thus, the equivalency of blood loss observed between the two groups in this study could be attributed to the high levels of expertise and experience in CLS for rectal cancer at our hospital.

In the present study the postoperative complication rates (CD grade ≥ II) in the RALS and CLS groups were 16.7% and 29.5% for the overall cohort, respectively. These results are comparable to previously reported rates of 8.9% to 33.1% and 18.4% to 31.7% for RALS and CLS, respectively.^7^, ^9^, ^31^, ^32^, ^33^, ^34^ In our study the postoperative complication rates (CD grade ≥ II) in the RALS group were significantly lower than those in the CLS group. The frequency of anastomotic leakage was 11.6% in the overall cohort (RALS 11.2% vs CLS 16.1%) and 12.2% in the matched cohort (RALS 10.4% vs CLS 21.4%). There was also a tendency for anastomotic leakage rates to be lower in the RALS group compared to the CLS group in the matched cohort (*p* = 0.0658). Previous studies reported anastomotic leakage rates of 1.5%–12.2% in patients who underwent RALS and 1.8%–10.4% in patients who underwent CLS.^7^, ^9^, ^31^, ^32^, ^33^, ^34^ In our study, although the incidence of anastomotic leakage in the RALS group was similar to those of previous reports, the leakage rates in the CLS group were relatively high compared to those published previously. Recent studies including propensity score‐matched analysis and meta‐analysis have shown the potential benefits of intraoperative ICG imaging for decreasing anastomotic leakage in MIS for rectal cancer.^35^, ^36^, ^37^ In the present study the rates of intraoperative blood flow assessed using ICG were significantly higher for both cohorts in the RALS group compared to the CLS group (*p* < 0.0001). One of the reasons for the difference in the incidence of anastomotic leakage between groups could be the significant differences observed in the results of the intraoperative blood flow test with ICG between groups.

The frequency of urinary retention in the overall and matched cohorts of the present study was 5.9% (RALS 1.7% vs CLS 8.7%) and 5.9% (RALS 1.1% vs CLS 10.6%), respectively. The incidence of urinary retention in the RALS group was significantly lower than that in the CLS group for both cohorts. Moreover, for both cohorts the days to soft diet and postoperative hospital stay in the RALS group were significantly shorter than those in the CLS group. Previous studies have reported on the potential benefits of RALS, including the lower complication rate, shorter postoperative hospital stay, and more favorable functional results, and several factors have been proposed to clarify why RALS is more advantageous than CLS for rectal cancer.^8^, ^9^, ^10^, ^12^, ^22^, ^23^, ^24^, ^26^ The wristed instruments facilitate ambidextrous capabilities and intuitive manipulation by surgeons, the camera provides stable, 3D, high‐definition imaging, while the robotic arm provides steady retraction and exposure. The combination of these attributes of robotic systems facilitates accurate anatomical dissection in the deep and narrow pelvis and could improve the preservation of autonomic pelvic functions.

The pathological findings of resection margin and the number of lymph nodes harvested indicate the high level of surgical and oncological quality of this method for rectal cancer. For the overall cohort, no statistically significant differences among DM, positive RM, and the number of lymph nodes harvested were observed. On the other hand, for the matched cohort the number of lymph nodes harvested by RALS was significantly higher than that in CLS, and there was a tendency for the positive‐RM rate in the RALS group to be lower than that in the CLS group. The results of the higher number of lymph nodes harvested in RALS group could contribute to the differences in the number of lateral lymph nodes harvested between groups. Because, although there were no significant differences in the number of lymph nodes harvested without LLND between the RALS and CLS group in the matched cohorts (15.5 vs 13.5, *p* = 0.1111), the number of lymph nodes harvested with the LLND in RALS group tended to be higher compared with those in the CLS group (29.6 vs 20.8, *p* = 0.0997). The differences of lateral harvested lymph nodes between groups might be associated with the procedures in the deep pelvis with technical complexity, such as LLND by using limited dexterity of instruments with fixed tips in CLS. Our pathological findings showed that the surgical quality of the RALS approach was comparable to, or better than, the CLS approach. However, further studies are necessary to evaluate other pathological parameters, such as CRM, which is an important predictor of oncological prognosis.^38^

This study has several limitations. First, this study has a nonrandomized and of retrospective design. To overcome this limitation, we performed case‐matched analysis using several clinical variables. The findings showed that the groups were well balanced and selection bias was mitigated. Second, this cohort study was performed at a single center and the size of the cohorts was relatively limited. As a result, the number of cases might be insufficient to draw definitive conclusions. Third, our propensity score‐matched analysis considered mainly baseline characteristics, but not factors such as surgeon experience and learning curve. Fourth, we did not assess sexual function and defecation function, which are essential for evaluating the clinical benefits of a treatment modality. Finally, as pathological parameters for assessing surgical and oncological quality, we evaluated DM and RM but not CRM. Consequently, our pathological analysis might be inadequate for assessing the completeness of TME.

In conclusion, the present propensity score‐matched analysis suggested that RALS is both safe and feasible for patients with rectal cancer. The results showed that the operative and pathological outcomes of RALS might be better than, or similar to, those of CLS. Further research is required to clarify whether the long‐term oncological outcomes are equivalent.

## AUTHOR CONTRIBUTIONS

Takahiro Yamanashi, Hirohisa Miura, Toshimichi Tanaka, Akiko Watanabe, Takuya Goto, Keigo Yokoi, and Ken Kojo contributed to data collection and analysis. Masahiro Niihara, Kei Hosoda, and Takashi Kaizu contributed to article preparation. Keishi Yamashita, Takeo Sato, Yusuke Kumamoto, Naoki Hiki, and Takeshi Naitoh contributed to the revision of the draft. All authors approved the final article for publication.

## CONFLICT OF INTEREST

The authors have no conflicts of interest to declare.

## ETHICS STATEMENT

This study was approved by the Ethics Committee of Kitasato University Hospital (Approval No.: B21‐071) and it conforms to the provisions of the Declaration of Helsinki.

## Data Availability

The data that support the findings of this study are available from the corresponding author upon reasonable request.

## References

[1] Kang SB , Park JW , Jeong SY , et al. Open versus laparoscopic surgery for mid or low rectal cancer after neoadjuvant chemoradiotherapy (COREAN trial): short‐term outcomes of an open‐label randomised controlled trial. Lancet Oncol. 2010;11:637‐645. 20610322 10.1016/S1470-2045(10)70131-5

[2] van der Pas MH , Haglind E , Cuesta MA , et al. Laparoscopic versus open surgery for rectal cancer (COLOR II): short‐term outcomes of a randomised, phase 3 trial. Lancet Oncol. 2013;14:210‐218. 23395398 10.1016/S1470-2045(13)70016-0

[3] Jeong SY , Park JW , Nam BH , et al. Open versus laparoscopic surgery for mid‐rectal or low‐rectal cancer after neoadjuvant chemoradiotherapy (COREAN trial): survival outcomes of an open‐label, non‐inferiority, randomised controlled trial. Lancet Oncol. 2014;15:767‐774. 24837215 10.1016/S1470-2045(14)70205-0

[4] Bonjer HJ , Deijen CL , Abis GA , et al. A randomized trial of laparoscopic versus open surgery for rectal cancer. N Engl J Med. 2015;372:1324‐1332. 25830422 10.1056/NEJMoa1414882

[5] Fleshman J , Branda M , Sargent DJ , et al. Effect of laparoscopic‐assisted resection vs open resection of stage II or III rectal cancer on pathologic outcomes: the ACOSOG Z6051 randomized clinical trial. JAMA. 2015;314:1346‐1355. 26441179 10.1001/jama.2015.10529 PMC5140087

[6] Stevenson AR , Solomon MJ , Lumley JW , et al. Effect of laparoscopic‐assisted resection vs open resection on pathological outcomes in rectal cancer: the ALaCaRT randomized clinical trial. JAMA. 2015;314:1356‐1363. 26441180 10.1001/jama.2015.12009

[7] Jayne D , Pigazzi A , Marshall H , et al. Effect of robotic‐assisted vs conventional laparoscopic surgery on risk of conversion to open laparotomy among patients undergoing resection for rectal cancer: the ROLARR randomized clinical trial. JAMA. 2017;318:1569‐1580. 29067426 10.1001/jama.2017.7219 PMC5818805

[8] Kim JY , Kim NK , Lee KY , Hur H , Min BS , Kim JH . A comparative study of voiding and sexual function after total mesorectal excision with autonomic nerve preservation for rectal cancer: laparoscopic versus robotic surgery. Ann Surg Oncol. 2012;19:2485‐2493. 22434245 10.1245/s10434-012-2262-1

[9] Yamaguchi T , Kinugasa Y , Shiomi A , Tomioka H , Kagawa H , Yamakawa Y . Robotic‐assisted vs conventional laparoscopic surgery for rectal cancer: short‐term outcomes at a single center. Surg Today. 2016;46:957‐962. 26482845 10.1007/s00595-015-1266-4

[10] Kim HJ , Choi GS , Park JS , Park SY , Yang CS , Lee HJ . The impact of robotic surgery on quality of life, urinary and sexual function following total mesorectal excision for rectal cancer: a propensity score‐matched analysis with laparoscopic surgery. Colorectal Dis. 2018;20:O103‐O113. 29460997 10.1111/codi.14051

[11] Kim MJ , Park SC , Park JW , et al. Robot‐assisted versus laparoscopic surgery for rectal cancer: a phase II open label prospective randomized controlled trial. Ann Surg. 2018;267:243‐251. 28549014 10.1097/SLA.0000000000002321

[12] Matsuyama T , Endo H , Yamamoto H , et al. Outcomes of robot‐assisted versus conventional laparoscopic low anterior resection in patients with rectal cancer: propensity‐matched analysis of the National Clinical Database in Japan. BJS Open. 2021;5(5):zrab083. 10.1093/bjsopen/zrab083 PMC8458638 34553225

[13] Brierley JDGM , Wittekind C . TNM Classification of Malignant Tumors. 8th ed. Wiley Blackwell; 2017.

[14] Miura H , Sato T , Tanaka T , et al. Short‐term outcomes of robot‐assisted surgery for rectal cancer. Kitasato Med J. 2020;50:152‐157.

[15] Japanese Society for Cancer of the Colon and Rectum . General Rules for Clinical and Pathological Studies on Cancer of the Colon, Rectum, and Anus. 6th ed. Kanehara & Co; 1998.

[16] Sato T , Kokuba Y , Koizumi W , Hayakawa K , Okayasu I , Watanabe M . Phase I trial of neoadjuvant preoperative chemotherapy with S‐1 and irinotecan plus radiation in patients with locally advanced rectal cancer. Int J Radiat Oncol Biol Phys. 2007;69:1442‐1447. 17855009 10.1016/j.ijrobp.2007.05.081

[17] Sato T , Ozawa H , Hatate K , et al. A phase II trial of neoadjuvant preoperative chemoradiotherapy with S‐1 plus irinotecan and radiation in patients with locally advanced rectal cancer: clinical feasibility and response rate. Int J Radiat Oncol Biol Phys. 2011;79:677‐683. 21035953 10.1016/j.ijrobp.2009.11.007

[18] Watanabe T , Muro K , Ajioka Y , et al. Japanese Society for Cancer of the colon and Rectum (JSCCR) guidelines 2016 for the treatment of colorectal cancer. Int J Clin Oncol. 2018;23:1‐34. 28349281 10.1007/s10147-017-1101-6 PMC5809573

[19] Dindo D , Demartines N , Clavien PA . Classification of surgical complications: a new proposal with evaluation in a cohort of 6336 patients and results of a survey. Ann Surg. 2004;240:205‐213. 15273542 10.1097/01.sla.0000133083.54934.ae PMC1360123

[20] Chan AC , Poon JT , Fan JK , Lo SH , Law WL . Impact of conversion on the long‐term outcome in laparoscopic resection of colorectal cancer. Surg Endosc. 2008;22:2625‐2630. 18297346 10.1007/s00464-008-9813-3

[21] Law WL , Poon JT , Fan JK , Lo SH . Comparison of outcome of open and laparoscopic resection for stage II and stage III rectal cancer. Ann Surg Oncol. 2009;16:1488‐1493. 19290491 10.1245/s10434-009-0418-4

[22] Xiong B , Ma L , Huang W , Zhao Q , Cheng Y , Liu J . Robotic versus laparoscopic total mesorectal excision for rectal cancer: a meta‐analysis of eight studies. J Gastrointest Surg. 2015;19:516‐526. 25394387 10.1007/s11605-014-2697-8

[23] Sun Y , Xu H , Li Z , et al. Robotic versus laparoscopic low anterior resection for rectal cancer: a meta‐analysis. World J Surg Oncol. 2016;14:61. 26928124 10.1186/s12957-016-0816-6 PMC4772524

[24] Li X , Wang T , Yao L , et al. The safety and effectiveness of robot‐assisted versus laparoscopic TME in patients with rectal cancer: a meta‐analysis and systematic review. Medicine (Baltimore). 2017;96:e7585. 28723798 10.1097/MD.0000000000007585 PMC5521938

[25] Prete FP , Pezzolla A , Prete F , et al. Robotic versus laparoscopic minimally invasive surgery for rectal cancer: a systematic review and meta‐analysis of randomized controlled trials. Ann Surg. 2018;267:1034‐1046. 28984644 10.1097/SLA.0000000000002523

[26] Kang J , Yoon KJ , Min BS , et al. The impact of robotic surgery for mid and low rectal cancer: a case‐matched analysis of a 3‐arm comparison—open, laparoscopic, and robotic surgery. Ann Surg. 2013;257:95‐101. 23059496 10.1097/SLA.0b013e3182686bbd

[27] Sng KK , Hara M , Shin JW , Yoo BE , Yang KS , Kim SH . The multiphasic learning curve for robot‐assisted rectal surgery. Surg Endosc. 2013;27:3297‐3307. 23508818 10.1007/s00464-013-2909-4

[28] Kim HJ , Choi GS , Park JS , Park SY . Multidimensional analysis of the learning curve for robotic total mesorectal excision for rectal cancer: lessons from a single surgeon's experience. Dis Colon Rectum. 2014;57:1066‐1074. 25101602 10.1097/DCR.0000000000000174

[29] Park EJ , Kim CW , Cho MS , et al. Multidimensional analyses of the learning curve of robotic low anterior resection for rectal cancer: 3‐phase learning process comparison. Surg Endosc. 2014;28:2821‐2831. 24902812 10.1007/s00464-014-3569-8

[30] Yamaguchi T , Kinugasa Y , Shiomi A , et al. Learning curve for robotic‐assisted surgery for rectal cancer: use of the cumulative sum method. Surg Endosc. 2015;29:1679‐1685. 25277477 10.1007/s00464-014-3855-5

[31] Cho MS , Baek SJ , Hur H , et al. Short and long‐term outcomes of robotic versus laparoscopic total mesorectal excision for rectal cancer: a case‐matched retrospective study. Medicine (Baltimore). 2015;94:e522. 25789947 10.1097/MD.0000000000000522 PMC4602485

[32] Serin KR , Gultekin FA , Batman B , et al. Robotic versus laparoscopic surgery for mid or low rectal cancer in male patients after neoadjuvant chemoradiation therapy: comparison of short‐term outcomes. J Robot Surg. 2015;9:187‐194. 26531198 10.1007/s11701-015-0514-3

[33] Huang YM , Huang YJ , Wei PL . Outcomes of robotic versus laparoscopic surgery for mid and low rectal cancer after neoadjuvant chemoradiation therapy and the effect of learning curve. Medicine (Baltimore). 2017;96:e8171. 28984767 10.1097/MD.0000000000008171 PMC5738003

[34] Law WL , Foo DCC . Comparison of short‐term and oncologic outcomes of robotic and laparoscopic resection for mid‐ and distal rectal cancer. Surg Endosc. 2017;31:2798‐2807. 27785627 10.1007/s00464-016-5289-8

[35] Li Z , Zhou Y , Tian G , et al. Meta‐analysis on the efficacy of Indocyanine green fluorescence angiography for reduction of anastomotic leakage after rectal cancer surgery. Am Surg. 2020;87:1910‐1919. 10.1177/0003134820982848 33377797

[36] Watanabe J , Ishibe A , Suwa Y , et al. Indocyanine green fluorescence imaging to reduce the risk of anastomotic leakage in laparoscopic low anterior resection for rectal cancer: a propensity score‐matched cohort study. Surg Endosc. 2020;34:202‐208. 30877565 10.1007/s00464-019-06751-9

[37] Yanagita T , Hara M , Osaga S , et al. Efficacy of intraoperative ICG fluorescence imaging evaluation for preventing anastomotic leakage after left‐sided colon or rectal cancer surgery: a propensity score‐matched analysis. Surg Endosc. 2021;35:2373‐2385. 33495878 10.1007/s00464-020-08230-y

[38] Kysters M , Marijnen CA , van de Velde CJ , et al. Patterns of local recurrence in rectal cancer; a study of the Dutch TME trial. Eur J Surg Oncol. 2010;36:470‐476. 20096534 10.1016/j.ejso.2009.11.011

